# Treatment-Related Risk Factors for Adverse Outcomes of COVID-19 in Patients Treated for Lymphoid Malignancies in the Pre-Omicron Era—A Study of KroHem, the Croatian Group for Hematologic Diseases

**DOI:** 10.3390/biomedicines12020331

**Published:** 2024-01-31

**Authors:** Igor Aurer, Ozren Jakšić, Sandra Bašić-Kinda, Stefan Mrđenović, Slobodanka Ostojić-Kolonić, Dominik Lozić, Hrvoje Holik, Sabina Novaković-Coha, Petra Berneš, Ivan Krečak, Martina Morić-Perić, Marino Narančić, Zdravko Mitrović, Toni Valković

**Affiliations:** 1 University Hospital Centre Zagreb, Kišpatićeva 12, 10000 Zagreb, Croatia; sandra.kinda@gmail.com; 2 School of Medicine, University of Zagreb, Šalata 3, 10000 Zagreb, Croatia or ozren.jaksic@mef.hr (O.J.); slobodankaostojic260@gmail.com (S.O.-K.); or zdravko.mitrovic@mef.hr (Z.M.); 3 University Hospital Dubrava, Av. G. Šuška 6, 10000 Zagreb, Croatia; 4 University Hospital Centre Osijek, J. Huttlera 4, 31000 Osijek, Croatia; mrdenovic@gmail.com; 5 Medical School, University of Osijek, J. Huttlera 4, 31000 Osijek, Croatia; 6 University Hospital Merkur, Zajčeva 19, 10000 Zagreb, Croatia; 7 University Hospital Centre Split, Spinčićeva 1, 21000 Split, Croatia; dlozic@kbsplit.hr; 8 General Hospital Dr. Josip Benčević, A. Štampara 42, 35000 Slavonski Brod, Croatia; hholik@gmail.com; 9 University Hospital Centre Sisters of Mercy, Vinogradska c. 29, 10000 Zagreb, Croatia; snovakovic89@gmail.com; 10 General Hospital Pula, Santoriova ul. 24a, 52100 Pula, Croatia; petrabernes@yahoo.com; 11 General Hospital Šibenik, S. Radića 83, 22000 Šibenik, Croatia; krecak.ivan@gmail.com; 12 Faculty of Medicine, University of Rijeka, Braće Branchetta 20, 51000 Rijeka, Croatia; toni.valkovic@medri.uniri.hr; 13 General Hospital Zadar, B. Peričića 5, 23000 Zadar, Croatia; martina.moricperic@gmail.com (M.M.-P.); marinonarancic@gmail.com (M.N.); 14 University Hospital Centre Rijeka, Krešimirova 42, 51000 Rijeka, Croatia

**Keywords:** lymphoma, chronic lymphocytic leukemia, COVID-19, rituximab, obinutuzumab, purine analogues

## Abstract

Patients with lymphoid malignancies are at increased risk of death or prolonged infection due to COVID-19. Data on the influence of different antineoplastic treatment modalities on outcomes are conflicting. Anti-CD20 monoclonal antibodies increase the risk of prolonged infection. It is unclear whether this risk is affected by the choice of the antibody (rituximab vs. obinutuzumab). To elucidate the role of antineoplastic therapy on COVID-19 outcomes, KroHem collected data on patients with lymphoid malignancies diagnosed with COVID-19 between October 2020 and April 2021. A total of 314 patients were identified, 75 untreated, 61 off treatment and 178 on treatment. The mortality rate in untreated and off-treatment patients was 15% and 16%; 9% and 10% had prolonged infection. In the on-treatment group, 3% were still prolonged positive at time of data collection, 62% recovered and 35% died; 42% had prolonged infection. Disease type, use of anti-CD20 monoclonal antibodies, prior autologous stem-cell transplantation (ASCT) and line of treatment did not significantly affect mortality. Mortality was higher in older patients (*p* = 0.0078) and those treated with purine analogues (*p* = 0.012). Prolonged COVID-19 was significantly more frequent in patients treated with anti-CD20 monoclonal antibodies (*p* = 0.012), especially obinutuzumab, and purine analogues (*p* = 0.012). Age, prior ASCT and treatment line did not significantly affect risk of prolonged infection. These data suggest that increased age and use of purine analogues are main risk factors for increased mortality of COVID-19 in patients with lymphoid malignancies. Obinutuzumab further increases the risk of prolonged disease, but not of death, in comparison to rituximab. Epidemiological considerations should be taken into account when choosing the appropriate antineoplastic therapy for patients with lymphoid malignancies.

## 1. Introduction

Lymphoid malignancies are a number of different entities with very variable clinical presentation, biology, response to therapy and prognosis, comprising one of the ten most frequent cancer types everywhere in the world [1]. While classifications of lymphoid neoplasms regularly change, basically they can be divided into B-cell malignancies, T/NK-cell malignancies and Hodgkin lymphomas. B-cell malignancies and T/NK cell malignancies can further present as either indolent or aggressive neoplastic diseases. These factors influence prognosis and therapeutic options. Indolent neoplasms do not need treatment until they show signs of progression or cause symptoms. Front-line therapy of lymphomas usually consists of standard-dose chemotherapy, combined with anti-CD20 monoclonal antibodies in the case of B-cell malignancies. Salvage therapy of aggressive lymphomas usually comprises the use of high-dose chemotherapy, with or without anti-CD20 monoclonal antibodies, and autologous stem-cell transplantation in responding patients.

In the early phases of the COVID-19 pandemic, the clinical course of the infection was severe and the mortality was high. A number of risk factors were identified resulting in more severe disease and dire outcomes, mainly related to age and metabolic disturbances [2]. Patients with lymphoid malignancies were a group with one of the highest mortality rates and frequency of prolonged infection [3,4,5]. This is considered to be a consequence of both underlying disease biology and treatment. B-cell depletion caused by unconjugated anti-CD20 monoclonal antibodies was soon identified as an important risk factor for the prolongation of the infection [6,7], but data on their effect on infection lethality are conflicting. Some studies suggested that anti-CD20 monoclonal antibodies also increase mortality [6,8], while some did not [9,10,11,12,13]. With two exceptions [9,14], all studies suggested that patients on treatment have inferior outcomes than those who did not start or finished therapy more than three to six months ago. However, few of the published studies examined in detail the effect of specific antineoplastic therapies, such as different cytotoxic agents and/or targeted drugs.

Purine analogues: fludarabine, bendamustine and cladribine were widely used at the onset of the pandemic in the treatment of chronic lymphocytic leukemia, indolent B-cell non-Hodgkin lymphomas and mantle-cell lymphoma [15,16]. In these disease types, they are more effective than alternative chemotherapeutic options but result in prolonged T- and B-cell lymphodepletion and increase the risk of late infections after end of treatment [17,18].

Two types of unconjugated anti-CD20 monoclonal antibodies are currently used for treatment of B-cell malignancies: rituximab and obinutuzumab. The latter has increased antibody-dependent cellular cytotoxicity (ADCC) and reduced complement-dependent cytotoxicity (CDC) in comparison with the former and results in faster, deeper and longer B-cell depletion albeit coupled with more hematological toxicity [19]. Obinutuzumab has been shown to be more effective than rituximab in follicular lymphoma and chronic lymphocytic leukemia, but not in non-follicular indolent non-Hodgkin lymphomas and aggressive B-cell lymphomas [20,21,22]. The published data comparing outcomes of patients treated with different anti-CD20 monoclonal antibodies who developed COVID-19 is very limited. A single study suggested that patients treated with obinutuzumab have worse outcomes than those treated with rituximab [23].

In order to more precisely describe outcomes of COVID-19 infection in patients with lymphoid malignancies and elucidate prognostic factors, especially the effects of different disease types, types of antineoplastic agents and anti-CD20 monoclonal antibodies on mortality and duration of COVID-19 in patients with mature lymphoid malignancies, KroHem, the Croatian Cooperative Group for Hematologic Diseases collected and analyzed data on outcomes of these patients who became infected in the pre-omicron era.

## 2. Materials and Methods

This was a retrospective, non-interventional study. Patients were included in the analysis if they were diagnosed with a mature lymphoid neoplasm prior to or at the time of COVID-19 diagnosis. The disease had to be diagnosed by standard criteria. Infection had to start at or before 30 April 2021 and had to be confirmed by a positive reverse transcriptase—polymerase chain reaction test. Duration of the disease was calculated from the date of symptom start until a negative polymerase chain reaction test or at least 2 weeks without symptoms. Data lock was in June 2021.

For the purpose of this analysis, lymphoid malignancies were grouped into: (1) indolent non-Hodgkin lymphomas, including follicular lymphoma, marginal zone lymphoma, lymphoplasmocytoid lymphoma and hairy-cell leukemia; (2) chronic lymphocytic leukemia; (3) mantle-cell lymphoma; (4) aggressive B-cell non-Hodgkin lymphomas, including B-large cell lymphomas, Burkitt lymphoma, and high-grade lymphomas; (5) Hodgkin lymphomas; (6) aggressive T-cell lymphomas, including peripheral T-cell lymphomas, anaplastic large T/NK-cell lymphomas and T-prolymphocytic leukemia; and (7) indolent T/NK-cell leukemias of the large granular lymphocyte type. Patients were considered untreated if they have not received any antineoplastic therapy for their lymphoid malignancy prior to infection start, on treatment if the period between last administration of therapy and infection was 3 months or less, and off treatment if that period was longer than 3 months. Antineoplastic regimens were grouped into (1) allogeneic stem cell transplantation and CAR-T cell (chimeric antigen T-cell) therapies; (2) purine analogues, including fludarabine, bendamustine and cladribine, alone or in combination with other antineoplastic agents; (3) high-dose chemotherapy (without purine analogues), including regimes such as DHAP (dexamethasone, cytarabine, cisplatin) or ICE (ifosfamide, carboplatin, etoposide); (4) standard-dose chemotherapy (without purine analogues), including CHOEP (cyclophosphamide, doxorubicine, vincristine, etoposide, steroid), DA-EPOCH (dose-adjusted cyclophosphamide, doxorubicine, vincristine, etoposide, steroid), CHOP (cyclophosphamide, doxorubicine, vincristine, steroid), CVP (cyclophosphamide, vincristine, steroid), chlorambucil, and similar; (5) the Bruton tyrosine kinase inhibitor ibrutinib; and (6) venetoclax. Patients were also divided according to whether they received anti-CD20 monoclonal antibodies or not. Those receiving more than one line of therapy prior to infection were grouped according to their last treatment line.

**Figure 1:**
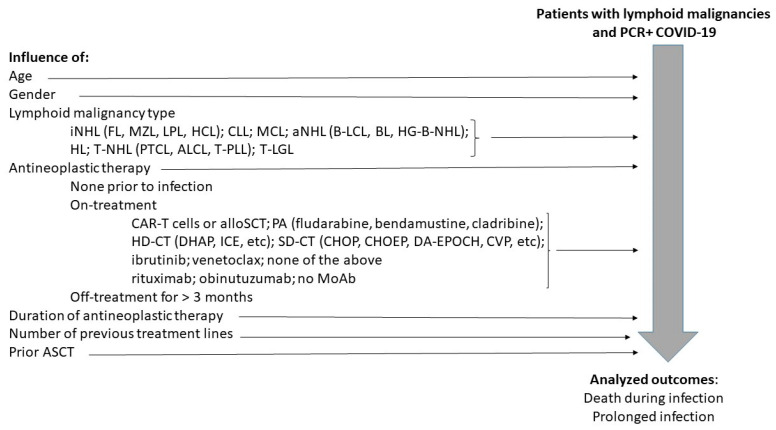
Study diagram. iNHL = indolent B-cell non-Hodgkin lymphomas; FL = follicular lymphoma; MZL = marginal zone lymphoma; LPL = lymphoplasmocytoid lymphoma; HCL = hairy-cell leukemia; CLL = chronic lymphocytic leukemija; MCL = mantle-cell lymphoma; aNHL = aggressive B-cell lymphomas, B-LCL = B-large cell lymphomas; BL = Burkitt lymphoma; HG-B-NHL = high-grade B-cell non-Hodgkin lymphomas; HL = Hodgkin lymphomas; T-NHL = aggressive T-cell lymphoma; PTCL = peripheral T-cell lymphomas; ALCL = T/NK anaplastic large cell lymphoma; T-PLL = T-cell prolymphocytic leukemia; T-LGL = T/NK cell large granular lymphocyte leukemia; CAR-T = chimeric antigen T-cell; alloSCT = allogeneic stem cell transplantation; PA = purine analogues; HD-CT = high-dose chemotherapy; SD-CT = standard-dose chemotherapy; MoAb = anti-CD20 monoclonal antibody; ASCT = autologous stem cell transplantation.

Analyzed outcomes included mortality rate and the frequency of prolonged infection. Death during infection was considered due to COVID-19. Patients were considered to have prolonged infection if they were continuously or repetitively positive by polymerase chain reaction test for more than 6 weeks or failed to clear symptoms for longer than 4 weeks. The percentage of patients with prolonged disease was calculated based on number of patients with available data alive 6 weeks after beginning of infection. A diagram of the study design is shown in Figure 1.

Statistical analysis was performed using freely available programs. Categorical variables were compared using the Fisher’s exact or χ^2^ test, numerical variables using the Mann–Whitney U test (https://www.socscistatistics.com/tests (accessed at 30 July 2021)) and MANOVA (multivariate analysis of variance) for multivariate analysis (SPSS Statistics, v.15).

This study was conducted according to the guidelines of the Declaration of Helsinki. No personal data were ever divulged. Since this was a retrospective study of anonymized patient data, informed consent was not required.

## 3. Results

Three hundred and fourteen patients fulfilled entry criteria (Table 1). They were between 20 and 88 years old (median 66), 180 were male and 134 female. A total of 75 were untreated, 61 were off treatment and 178 on treatment.

In the untreated group, 11 (15%) died; 10% had prolonged infection, none of whom died. In the off-treatment group, 10 (16%) died; 9% had prolonged infection, none of whom died.

In the on-treatment group, 6 (3%) were still prolonged positive at time of data lock, 110 (62%) recovered and 62 (35%) died; 42% had prolonged infection, of whom 47% recovered, 42% died and 11% were still positive at data lock.

The single allografted and both patients treated with CAR-T cells for B-large cell lymphoma died after prolonged infection. We analyzed prognostic factors for lethal and prolonged infection in the remaining 175 conventionally treated patients. Thirty-five of them received antineoplastic therapy without anti-CD20 monoclonal antibodies, 116 were treated with rituximab and 24 with obinutuzumab.

The differences in outcomes related to disease type failed to reach statistical significance (Table 2). None of the two patients with T/NK-cell large granular lymphocyte leukemia died or had prolonged infection. One of seven patients with Hodgkin lymphomas died, none had prolonged infection. These two groups seemed to have best outcomes. Conversely, patients with mantle-cell lymphoma seemed to have worst outcomes with a mortality rate of 55% and prolonged infection rate of 77%. The rate of prolonged COVID-19, but not mortality, seemed lower in patients with aggressive T-cell lymphomas than in other disease subtypes.

Age was significantly related to infection lethality (Table 3). Mortality in the oldest tercile (patients older than 70 years of age) was more than double in comparison to the youngest tercile (patients younger than 60 years of age) (43% vs. 19%). In contrast, age had no influence on the risk of prolonged infection.

**Figure 2:**
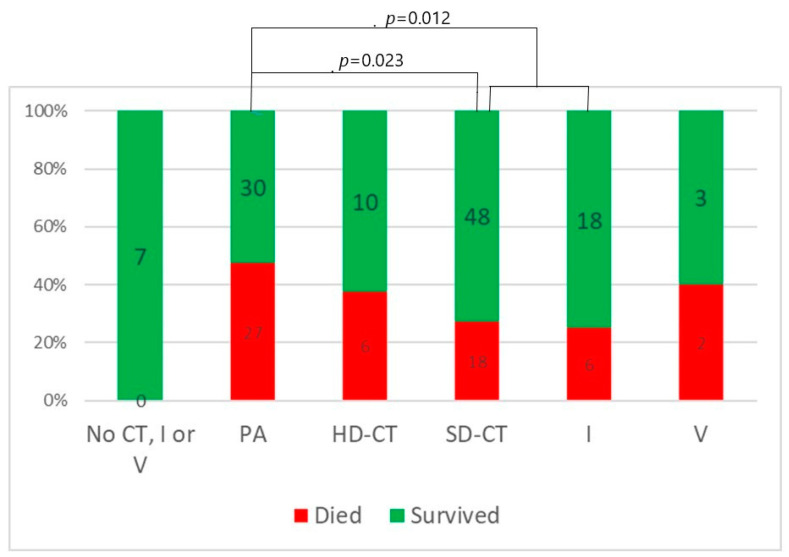
Mortality according to type of antineoplastic treatment. Numbers in bars indicate the number of patients. CT = chemotherapy; PA = purine analogues; HD-CT = high-dose chemotherapy; SD-CT = standard-dose chemotherapy; I = ibrutinib; V = venetoclax.

**Figure 3:**
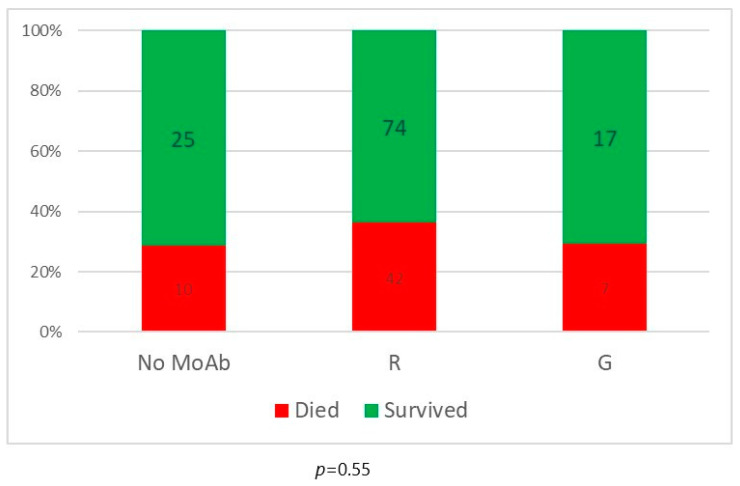
Mortality according to the use of anti-CD20 monoclonal antibodies (MoAb). Numbers in bars indicate the number of patients. R = rituximab; G = obinutuzumab.

**Figure 4:**
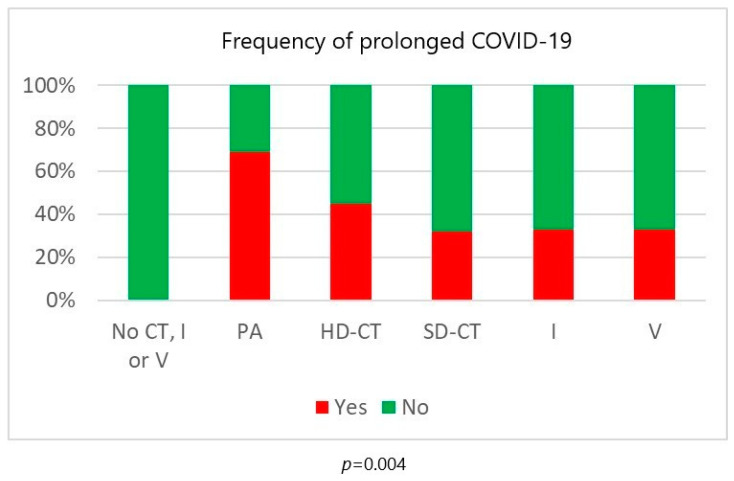
Risk of prolonged COVID-19 according to antineoplastic treatment. CT = chemotherapy; PA = purine analogues; HD-CT = high-dose chemotherapy; SD-CT = standard-dose chemotherapy; I = ibrutinib; V = venetoclax.

**Figure 5:**
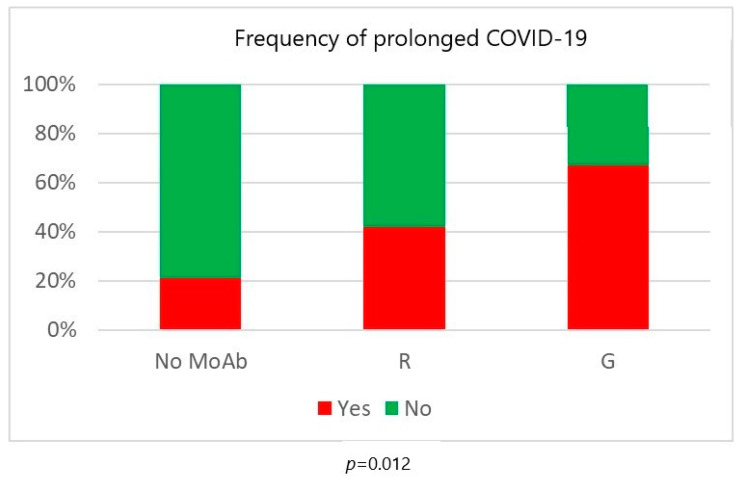
Risk of prolonged COVID-19 according to the use of anti-CD20 monoclonal antibodies (MoAb). R = rituximab; G = obinutuzumab.

**Figure 6:**
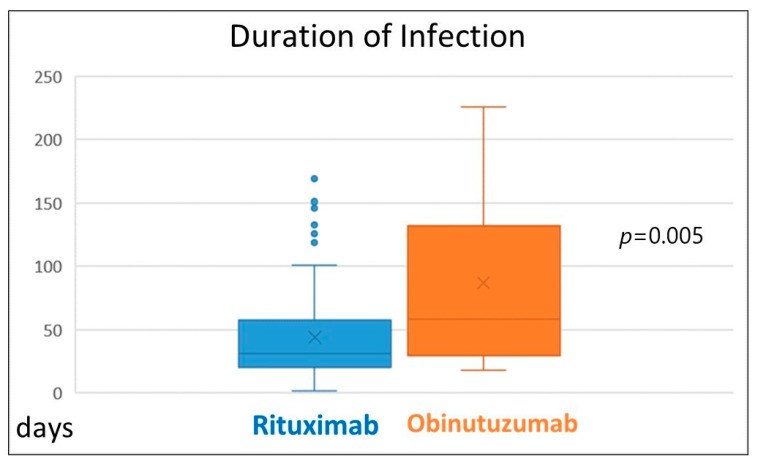
Infection duration in patients treated with rituximab and obinutuzumab.

Regarding treatment type, seven patients with indolent B-cell non-Hodgkin lymphomas and T/NK-cell large granular lymphocyte leukemia were treated with rituximab monotherapy, vemurafenib or cyclosporine without chemotherapy, ibrutinib or venetoclax. None of them died and none had prolonged infection. Patients treated with purine analogues had significantly higher mortality rates in comparison to those treated with standard-dose chemotherapy or ibrutinib (47% vs. 27% vs. 25%, *p* = 0.012). Infection lethality in those treated with high-dose chemotherapy (38%) and venetoclax (40%) was intermediate (Figure 2). Mortality of patients treated with and without anti-CD20 monoclonal antibodies was similar (Figure 3). Lethal COVID-19 occurred in 29% of patients treated without anti-CD20 monoclonal antibodies, 36% of those treated with rituximab and 29% treated with obinutuzumab (*p* = 0.55). Prolonged infection was more frequent in patients treated with purine analogues (69% vs. 28%, *p* = 0.004) (Figure 4) or anti-CD20 monoclonal antibodies (21%, in patients treated without anti-CD20 monoclonal antibodies, 42% in those treated with rituximab 42%, and 67% in those treated with obinutuzumab; *p* = 0.012) (Figure 5). Duration of infection was significantly longer in patients treated with obinutuzumab than in those treated with rituximab (Figure 6). The effects of treatment type were independent of age.

Number of cycles or duration of treatment were not significantly related to mortality or risk of prolonged infection. Patients on maintenance therapy with anti-CD20 monoclonal antibodies after immunochemotherapy induction had outcomes similar to those of patients who developed COVID-19 during induction. Gender, number of treatment lines, previous autologous stem cell transplantation and use of antiviral therapy did not significantly affect neither mortality nor the rate of prolonged infection.

## 4. Discussion

Patients included in our series were treated during the early phases of the COVID-19 pandemic characterized by viral strains causing severe disease in immunocompetent and even more so in severely immunocompromised hosts. At that time, adequately controlled studies of antiviral therapy did not exist, recommendations on their use were frequently contradictory, and the only available antiviral agent, introduced only during the duration of our study, was remdesivir [24]. In many centers, this drug was administered only in patients who developed severe COVID-19 or not used at all. The same was true for convalescent fresh-frozen plasma. This probably explains why there was no consistent effect of antiviral therapy on outcomes. Vaccination against COVID-19 started in Croatia in December 2020, and most patients received the second dose in the beginning of 2021. Since the majority of patients on treatment for lymphoid neoplasms fail to raise adequate humoral responses [25,26,27,28,29], it seems reasonable to conclude that almost all of the analyzed patients were unprotected against the infection. All of this explains the high mortality rate, different from the current situation, but similar to that of other contemporary series [3,4].

The results presented in this paper are in accordance with the generally accepted attitude that patients with lymphoid neoplasms are more sensitive to COVID-19 than the general population, even before they start antineoplastic therapy. The mortality rate in previously untreated patients was 15%, which is significantly higher than the 2.2% in the general Croatian population at that time [30,31]. The same is true for the tendency to develop prolonged infection (10% vs. <0.1%). This sensitivity is significantly increased by antineoplastic therapy, resulting in an increase in mortality to 35% and frequency of prolonged infection to 42%.

In accordance with other series, the mortality was highest in the very small group of patients becoming infected within 3 months from receiving an allogeneic stem cell transplant or CAR-T cell therapy, during the period in which their immune system has still not began to recover from procedure-related damage [32,33,34].

However, the results of this analysis suggest that different conventional antineoplastic therapy options confer substantial differences in sensitivity to COVID-19. Patients treated with purine analogues had the highest mortality rate followed by high-dose chemotherapy, standard-dose chemotherapy and ibrutinib. The number of patients receiving venetoclax was too small to draw meaningful conclusions. Purine analogues were also the only type of chemotherapy that increased the risk of prolonged COVID-19. The addition of anti-CD20 monoclonal antibodies to chemotherapy did not increase mortality over that of chemotherapy alone, but prolonged the duration of chemotherapy-induced sensitivity to COVID-19 (as witnessed by the fact that patients who developed COVID-19 during maintenance did not have better outcomes than those who developed the infection during induction) and significantly increased the risk of prolonged infection. Since, of all the chemotherapy options, purine analogues have the strongest and longest lymphodepleting effect on both B and T cells, these data support the hypothesis that the outcome of infection is primarily dependent on host T-cell function and the duration of infection on B-cell function [7,35].

The results of this study are not completely consistent with those of other published studies, but very few of them examined in detail effects of different types of chemotherapy. Those that did, did find signs of detrimental effects of, e.g., immunochemotherapy in patients with chronic lymphocytic leukemia (which at that time was mostly based on purine analogues) [12] and use of bendamustine in patients with lymphoma [9]. The single study reporting differences in outcome between rituximab and obinutuzumab was smaller than ours, conducted in a different time-point, i.e., in a period when the omicron strain became most prevalent, and did not try to separate the effects of anti-CD20 monoclonal antibodies from those of chemotherapy [23].

In our analysis, patients were considered to be on treatment if the interval between the last application of antineoplastic therapy and start of infection was three months or less. This period is somewhat arbitrary and has been chosen to replicate other similar studies. The data collected and available for analysis were insufficient to ascertain whether the increased sensitivity to COVID-19 lasts 3, 6 or 12 months after the end of antineoplastic therapy. However, it seems that within this timeframe, the sensitivity to COVID-19 diminishes to a level similar to that of untreated patients, both in terms of mortality and risk of prolonged infection.

As in all other published studies, age was the most important unmodifiable risk factor for mortality. Mantle-cell lymphoma is a lymphoma type preferentially affecting elderly men and is treated with anti-CD20 monoclonal antibodies and purine analogue-containing chemotherapy, which probably explains the fact that this disease type had worst outcomes of all, both related to mortality and frequency of prolonged infection [36].

The differences in outcomes between different disease subtypes failed to reach statistical significance and can be explained by differences in antineoplastic therapy and age. Mortality was less in patients treated without chemotherapy (e.g., T/NK-cell large granular lymphocyte leukemia patients), with less intensive chemotherapy regimens (e.g., patients with indolent B-cell non-Hodgkin lymphomas) or younger (e.g., Hodgkin lymphoma patients). Rates of prolonged infection were lowest in patients treated without purine analogues and anti-CD20 monoclonal antibodies (e.g., those with T/NK-cell large granular lymphocyte leukemias, Hodgkin lymphomas or aggressive T-cell lymphomas).

Despite the change in severity of the infection occurring in the later phase of the pandemic, our data can be interesting also for the present situation. Prolonged COVID-19, which is still a problem, interferes with regular antineoplastic treatment of patients, reducing its efficacy and expected cure and survival rates. Also, the elucidation of prognostic factors might shed additional light into the biology of the infection and host defense mechanisms.

This study has many limitations, mainly related to its design. All participating centers tried to collect data on all consecutive patients, but the possibility remains that some died at home or at other hospitals and were not included in this data set. Other risk factors, such as obesity, diabetes and smoking, could not be analyzed in detail, but they seem to be of lesser importance in patients treated for hematological malignancies. Also, due to the non-randomized retrospective, real-life nature of the data, and relatively small numbers of patients in each category it is impossible to draw firm conclusions on causality. Still, the presented data are interesting and possibly important for making informed clinical decisions on therapy choices but also add to the totality of evidence on biological defense mechanisms against COVID-19.

## 5. Conclusions

Use of purine analogues, such as bendamustine and fludarabine, should probably be avoided in patients with indolent B-cell non-Hodgkin lymphomas and chronic lymphocytic leukemia, diseases for whom other effective treatments are available, during the COVID-19 pandemic. Anti-CD20 monoclonal antibodies seem to have a smaller effect on mortality than chemotherapy, with obinutuzumab increasing the risk of prolonged disease, but not of death, more than rituximab.

## Figures and Tables

**Table 1:** Number of patients according to disease type.

Disease Type	iNHL	CLL	aNHL	MCL	LGL	T-NHL	HL	Total
Untreated (no.)	17	44	10	1	1	0	2	75
Off treatment (no.)	13	19	15	0	0	2	12	61
On treatment (no.)	57	39	41	22	2	10	7	178

iNHL = indolent B-cell lymphoma; CLL = chronic lymphocytic leukemia; aNHL = aggressive B-cell lymphoma; LGL = indolent T/NK cell leukemia; T-NHL = aggressive T-cell leukemia/lymphoma; HL = Hodgkin lymphomas.

**Table 2:** Outcome of patients on treatment according to disease type.

Disease Type	iNHL	CLL	aNHL	MCL	LGL	T-NHL	HL	Total	*p* Value
No.	57	39	41	22	2	10	7	**178**	
Mortality	26%	36%	41% *	55%	0	30%	14%	35% *	0.217
Prolonged infection	44%	39%	36% *	77%	0	13%	0	42% *	0.051

iNHL = indolent B-cell lymphoma; CLL = chronic lymphocytic leukemia; aNHL = aggressive B-cell lymphoma; LGL = indolent T/NK cell leukemia; T-NHL = aggressive T-cell leukemia/lymphoma; HL = Hodgkin lymphomas. * including one allografted and two patients treated with CAR-T cells.

**Table 3:** Outcome of patients on treatment according to age.

Age (Years)	<60	60–69	>70	*p* Value
No.	57	59	58	
Mortality	19%	39%	43%	0.008
Prolonged infection	40%	46%	38%	0.562

## Data Availability

Due to the informed consent waiver, any requests for original data should be sent to the corresponding author and must be approved by a Croatian Ethics Committee.
